# Canonical Wnt Signaling in the Pathology of Iron Overload-Induced Oxidative Stress and Age-Related Diseases

**DOI:** 10.1155/2022/7163326

**Published:** 2022-01-25

**Authors:** Austin Armstrong, Ashok Mandala, Milan Malhotra, Jaya P. Gnana-Prakasam

**Affiliations:** ^1^Department of Ophthalmology, Saint Louis University, St. Louis, USA; ^2^Department of Biochemistry & Molecular Biology, Saint Louis University, St. Louis, USA

## Abstract

Iron accumulates in the vital organs with aging. This is associated with oxidative stress, inflammation, and mitochondrial dysfunction leading to age-related disorders. Abnormal iron levels are linked to neurodegenerative diseases, liver injury, cancer, and ocular diseases. Canonical Wnt signaling is an evolutionarily conserved signaling pathway that regulates many cellular functions including cell proliferation, apoptosis, cell migration, and stem cell renewal. Recent evidences indicate that iron regulates Wnt signaling, and iron chelators like deferoxamine and deferasirox can inhibit Wnt signaling and cell growth. Canonical Wnt signaling is implicated in the pathogenesis of many diseases, and there are significant efforts ongoing to develop innovative therapies targeting the aberrant Wnt signaling. This review examines how intracellular iron accumulation regulates Wnt signaling in various tissues and their potential contribution in the progression of age-related diseases.

## 1. Introduction

A multitude of age-related diseases are associated with disrupted cellular iron homeostasis. Wnt/*β*-catenin signaling, also known as canonical Wnt signaling, is a crucial pathway that mediates cell development and proliferation. Hence, abnormal Wnt signaling can be detrimental and is implicated in diseases across different tissue types. Interestingly, an overlap between dysregulated iron homeostasis and aberrant Wnt signaling exists in pathologies including colorectal carcinoma (CRC) [1–3], diabetic retinopathy (DR) [4, 5], and age-related macular degeneration (AMD) [6–8]. For this reason, there has been a recent interest in studying the interplay between iron homeostasis and canonical Wnt signaling.

Iron is a necessary dietary component, as it is crucial to processes ranging from oxygen transport by red blood cells [9] to fetal neurodevelopment [10]. Cellular iron homeostasis and mitochondrial iron homeostasis are interdependent as mitochondria must import iron to form iron–sulfur clusters and heme, which are critical for vital cellular functions. Imbalances in iron homeostasis leading to its accumulation can be particularly dangerous, such as in hereditary hemochromatosis [11]. The toxicity due to iron overload is largely related to the potential of iron to induce oxidative stress, mitochondrial dysfunction, and inflammation. Iron-mediated oxidative stress arises because excess iron, a prooxidant, generates reactive oxygen species (ROS) via the Fenton reaction [12]. ROS can then affect the integrity of DNA [13], as well as other important proteins and lipids involved in cellular function [14]. Iron-mediated mitochondrial dysfunction is a result of iron-induced mitochondrial DNA damage, which correlates with defects in iron–sulfur cluster biogenesis, electron transport chain, and heme synthesis [15]. Iron-mediated inflammation occurs due to the proinflammatory nature of both excess labile iron [16] and iron bound to ferritin [17, 18], an iron storage protein. Cellular labile iron promotes inflammation via interleukin-1*β* (IL-1*β*) secretion and NLRP3 inflammasome stimulation in human monocytes through the NF-*κ*B pathway [16]. Similarly, ferritin has been shown to function as a local cytokine through NF-*κ*B pathway activation in activated rat stellate cells, leading to significant increases in inflammatory molecules such as IL-1*β* [17]. Hyperferritinemia, a condition characterized by excess ferritin levels, is constitutively linked to disorders like familial hemophagocytic lymphohistiocytosis [17] and macrophage activation syndrome [18] that are characterized by overactive inflammatory responses. In addition, research has demonstrated that inflammation itself can lead to iron accumulation in tissues such as the retina [19] and the liver [20] by upregulating hepcidin and thereby decreasing ferroportin expression. These findings indicate a positive feedback loop, where iron-mediated inflammation leads to further iron accumulation and subsequently increased iron-mediated inflammation, oxidative stress, and mitochondrial dysfunction.

The rationale for much of the research discussed in the following sections is that oxidative stress [21–23], inflammation [24, 25], and mitochondrial dysfunction [26] have also been indicated as mediators of abnormal canonical Wnt signaling. This overlap raises the fundamental question of our review: what role does iron overload have in regulating Wnt signaling? While the relationship between iron, Wnt signaling, and cancer has become increasingly well-established [27–29], this same relationship is less documented in pathologies outside of cancer. The present review is aimed at providing an update on the canonical Wnt pathway with respect to iron-mediated aberrant Wnt/*β*-catenin signaling in several tissue-specific contexts.

## 2. The Canonical Wnt Pathway

### 2.1. Contextualizing Wnt

The first Wnt gene was discovered in 1982 as an oncogene activated in mouse models of virally induced mammary carcinoma and was named Int-1 [30]. Several years later, an important gene in Drosophila larval development known as the wingless (Wg) gene was found to be a homolog of the murine Int-1 [31]. The discovery of the Wg homolog to mouse Int-1 and subsequent research [32, 33] has demonstrated that the canonical Wnt pathway is highly conserved amongst a variety of species. Even complexity within the Wnt gene family seems to be conserved, as the sea anemone *Nematostella vectensis* shares 11 of the 12 known Wnt gene subfamilies with humans [32]. Because of the conserved nature of the Wnt genes and the implication of Wnt regulation in a variety of physiological events, Wnt/*β*-catenin research has led to a plethora of discoveries over the last four decades.

Today, we know of 19 Wnt genes in the mammalian genome that encode 12 subfamilies of Wnt proteins (Wnts) [34]. These Wnts are involved in a range of cell events but centrally act as growth factors and cause cell proliferation in a variety of cell types during both embryogenesis and adult tissue homeostasis [35–37]. In addition, Wnts contribute to the directional organization of proliferating cells by altering gene expression and affecting cytoskeletal and mitotic architecture [38–43]. All Wnt genes encode glycoproteins that are about 40 kDA in size and that contain many conserved cysteine residues [44]. After translation, important palmitoylation and glycosylation events occur on Wnt proteins in the endoplasmic reticulum (ER). Palmitoylation involves the attachment of a palmitoleic acid to a conserved serine residue on Wnt proteins [45, 46] by an O-acyltransferase known as porcupine [47, 48]. This lipid modification is necessary for Wnt signaling, as it is used downstream as a binding site for the Wnt receptor, Frizzled [49], as well as the Wntless protein that aids in Wnt secretion [50, 51]. Of the two major types of posttranslational modifications in the ER, glycosylation is the one less understood. Wnts vary in the number of glycosylated sites present, and the presence of these glycans seems to have varying importance. Site-directed mutagenesis of specific glycosylated regions impairs secretion in certain Wnts [52], while in other Wnt isoforms, it does not seem to have any affect at all [53]. Though further studies are required to determine the exact function of posttranslational Wnt glycosylation, glycosylation currently has significance as a signal for the secretion of few specific Wnt glycoproteins.

### 2.2. Wnt Trafficking and Secretion

The trafficking and secretory pathways are two examples of conserved characteristics between the human Wnt proteins [54, 55]. As aforementioned, Wnt trafficking and secretion are dependent upon proper lipid modification by porcupine in the ER [47, 48]. This is because Wntless (Wls), a transmembrane protein, binds the palmitoleic acid in the ER for proper Wnt trafficking and subsequent Wnt release [50, 51, 56]. Indeed, knockouts of Wls or inhibitors of porcupine result in the accumulation of Wnt proteins in ER [57]. Once bound to Wnt, Wls will travel with Wnt through the Golgi and ultimately to the plasma membrane [50, 51, 56]. Here, Wls may act in one of the two ways. One possibility is that Wls can leave its respective Wnt glycoprotein and return to the trans-Golgi network [58–60]. This occurs via retrograde movement involving clathrin-mediated endocytosis and retromer retrieval mediated by a specialized ER-retrieval motif [50, 57, 61, 62]. Alternatively, Wls may be incorporated into the membrane of an exosome along with Wnt for secretion [63, 64].

The distance of Wnt secretion and subsequent signaling is a heavily debated topic, as there is evidence for Wnt targeting both local [34, 65] and distant [66, 67] cells. At the Wnt secreting membrane, varying events can occur depending on the desired distance of secretion. Wnts can be tethered for contact-dependent interactions with nearby cells [68] or used for longer range signaling either through expression on an exosome with Wntless requiring R-SNARE Ykt6 activity [69] or by binding to solubilizing molecules, such as Swim [66]. After Wnt trafficking and secretion, signaling occurs. The known Wnt pathways can be organized into single canonical Wnt pathway and two noncanonical Wnt pathways [37]. This review will focus on the canonical Wnt pathway, which involves the nuclear localization of active *β*-catenin, as this is the best studied pathway in relation to iron homeostasis.

### 2.3. Wnt/*β*-Catenin Signaling Cascade

In canonical Wnt signaling, there is a complex system of transducing factors that cooperate to instill cell responsiveness to the Wnt ligands. A central player in this cascade is *β*-catenin, as it is the primary regulator in Wnt target gene expression. With cytosolic accumulation of *β*-catenin, subsequent nuclear translocation occurs allowing the protein to bind to the T-Cell Factor/Lymphoid Enhancer Factor (TCF/LEF) transcription factor family [70, 71]. Wnt target cells exhibit the surface receptors Frizzled (FZD) and low-density lipoprotein-related receptor protein 5 or 6 (LRP5/6) to initiate the signal cascade [34]. FZD is a family of cell surface proteins with seven transmembrane domains that contain both an extracellular N-terminal cysteine-rich domain that interacts with Wnt [49, 72, 73], as well as a hydrophobic groove that binds the lipid modifications on Wnt [49]. LRP5 and LRP6 serve as coreceptors necessary for Wnt transduction that form heterodimers with FZD in the presence of Wnt [74, 75]. It has been posited that LRPs contain binding regions for Wnt, as described by several anti-LRP monoclonal antibody studies [76]. While there are a variety of other Wnt receptors that are important for noncanonical signaling [77], the FZD and LRP-mediated pathway will remain the focus of this review as it is the best understood with relation to iron.

In the absence of Wnt, cytoplasmic *β*-catenin is bound by an intact destruction complex and targeted for ubiquitin-mediated degradation, preventing *β*-catenin accumulation and nuclear translocation. This ultimately results in Wnt target gene repression. The destruction complex consists of proteins Axin, Adenomatous polyposis coli (APC), and Wilms tumor gene on the X chromosome (WTX), casein kinase 1-*α* (CK1*α*), glycogen synthase kinase 3-*β* (GSK-3*β*), and other factors [34]. Axin is a scaffolding protein that interacts with *β*-catenin and other members of the destruction complex [34]. APC and WTX, two tumor suppressor proteins, are also essential for an effective destruction complex [34]. CK1*α* and GSK-3*β* are serine-threonine kinases that perform multiple functions related to Wnt signaling. In terms of the destruction complex, they phosphorylate Axin-bound *β*-catenin to target it for ubiquitination [34, 78]. *β*-Transducin repeat-containing protein (*β*-TrCP), a component of the E3 ubiquitin ligase complex, recognizes phosphorylated *β*-catenin and catalyzes the addition of ubiquitin polymers to direct *β*-catenin for degradation by proteasomes preventing their nuclear translocation [78]. The absence of *β*-catenin in the nucleus leaves the repressive TCF/LEF complex active, which then permits Groucho/transducin-like enhancer (Gro/TLE) family proteins to recruit histone deacetylases that inhibit Wnt target gene expression [79].

**Figure 1:**
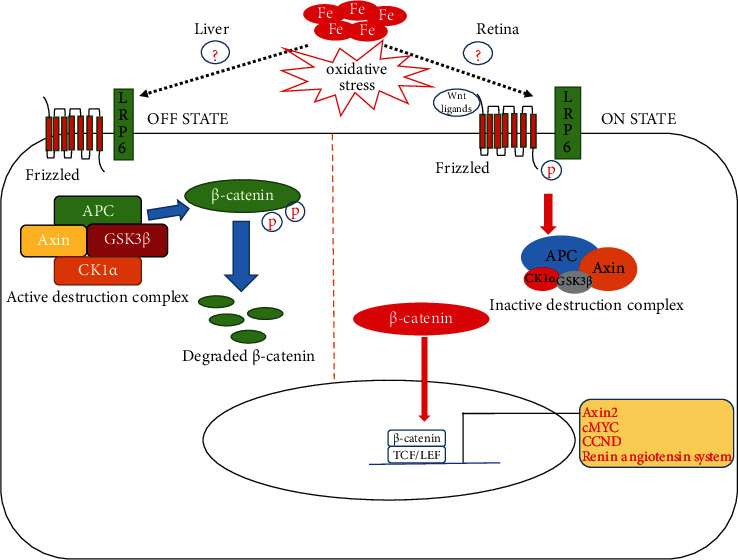
A schematic overview of tissue-dependent Wnt signaling during conditions of iron overload.

In the presence of Wnt, canonical Wnt stimulation begins with Wnt ligand facilitating the heterodimerization of FZD and its coreceptor, such as LRP5 or LRP6. Ligation induces a conformational change in the receptors and by a currently unclear mechanism, recruits, and activates GSK-3*β* and CK1*γ* [80–82]. At the membrane, GSK-3*β* and CK1*γ* phosphorylate the LRP tail at the PPPSP motif [83] and in regions adjacent to the PPPSP motifs [84], respectively. This sequential phosphorylation of the LRP tail results in Axin relocation to the plasma membrane and its physical removal from the cytosol, inhibiting the formation of the *β*-catenin destruction complex [34]. *β*-Catenin then accumulates in the cytosol and subsequently translocates to the nucleus. Nuclear *β*-catenin interacts with the TCF/LEF interface discussed earlier to activate Wnt target gene expression [70, 71, 85, 86] as shown in Figure 1.

### 2.4. Wnt/*β*-Catenin Modulation

As previously discussed, Wnt/*β*-catenin signaling plays a multitude of roles in a host of tissues throughout life. Wnts generally function as growth factors to cause proliferation and have more than 100 downstream target genes [35–37, 87]. Many of these target genes are important cell cycle regulators including C-Myc [88] and Cyclin D [89]. Others include those related to angiogenesis like VEGF [90], as well as nearly every component of the Renin-Angiotensin-System (RAS) [91]. Wnt signaling activates mitochondrial biogenesis, in turn producing elevated levels of ROS and oxidative damage [26], which is considered to be the cause for certain pathological consequences. Given the wide-ranging functions of canonical Wnt signaling, it is logical that dysfunctional Wnt/*β*-catenin signaling would have a variety of negative implications. The nature of this aberrance, however, is not as intuitive. In some diseases, such as in cases of CRC [92] or AMD [7, 8], Wnt signaling is pathologically upregulated, while in others, such as in Norrie disease [93] or osteopenia [94], Wnt signaling is pathologically downregulated. For this reason, a variety of positive and negative modulators exist and are important for the understanding of Wnt signaling.

#### 2.4.1. Positive Modulation

Positive modulators of Wnt/*β*-catenin signaling are those that lead to an increase in active *β*-catenin translocating to the nucleus and thus increasing the expression of Wnt target genes. In cases of chronic wounds [95], vitiligo [96, 97], or other diseases characterized by Wnt/*β*-catenin inactivation, positive Wnt modulators can be protective. However, in the context of diseases characterized by Wnt/*β*-catenin activation, positive Wnt modulators may be harmful. For example, patients being treated with lithium, a canonical Wnt activator, were shown to have a significantly increased chance of developing renal carcinogenesis [98].

One important positive regulation pathway of vertebrate Wnt signaling involves the four R-spondin (RSPO) proteins, which are characterized by two furin domains and a thrombospondin domain [99]. In the absence of RSPO ligands, the disheveled protein promotes the destruction of FZD on the membrane by the E3 ligases [100]. However, in the presence of RPSO proteins, the RSPO ligands will bind to leucine-rich repeat-containing G protein-coupled 5 (Lgr5) family receptors [101–103] resulting in the membrane clearance of E3 ligases RNF43 and ZNRF3 [104, 105]. In effect, this promotes FZD accumulation on the membrane and increases the sensitivity of a cell to Wnt ligands [104, 105]. Norrin is a second example of a secreted protein that positively modulates Wnt signaling [106]. It is characterized by its cysteine-knot motif [107] and, with the help of its coreceptor tetraspanin 12 [108, 109], acts by binding directly to the FZD4/LRP5 complex to activate the canonical Wnt pathway [93, 110, 111].

In addition to the well-studied RSPO and Norrin positive modulators, there are several others that have been discovered over the years. Protein phosphatase-2A (PP2A) is a serine-threonine phosphatase composed of three subunits that can positively regulate the canonical Wnt pathway by dephosphorylating a variety of proteins including *β*-catenin [112]. Other examples include microRNAs miR-135a and miR-135b that directly repress APC expression in colorectal cancer cells, thereby destabilizing the *β*-catenin destruction complex and allowing Wnt target gene expression [113]. Still, others like heparin sulfate proteoglycans serve as cofactors to promote Wnt signaling in the control of *C. elegans* mitotic spindle orientation [114], distal-tip cell migration [115], and neuronal positioning [116].

#### 2.4.2. Negative Modulation

Negative modulators of Wnt/*β*-catenin signaling are those that lead to the destruction of *β*-catenin and a decrease in the expression of Wnt target genes. This may be protective in diseases characterized by Wnt/*β*-catenin activation, but deleterious during diseases characterized by Wnt/*β*-catenin inactivation [117]. The potentially deleterious effect of negative modulators can be seen in the progression of Alzheimer's disease, a disease characterized by Wnt inactivation [118]. Specifically, the worsening of amyloid beta plaque accumulation and subsequent synaptic loss was observed with the presence of the negative regulator Dickkopf-1 [119, 120].

A prototypical downregulator of Wnt signaling is Notum, an extracellular enzyme that inhibits Wnt proteins by removing their vital palmitoylate residues [121, 122]. Notum also partakes in a negative feedback loop, as the TCF/LEF family transcription factors have binding sites in the Notum promoter region to increase its expression [121, 123]. In addition to Notum, extracellular Wnt inhibitors include Wnt inhibitory factor (WIF) proteins [124, 125] and secreted FZD-related proteins (SFRPs) [126–128] that directly bind Wnt proteins to inhibit signaling. There is also another important class of negative Wnt modulators that act as transmembrane antagonists by binding and subsequently blocking Wnt receptors from interacting with Wnt. These include Shisa [129], Wnt-activated inhibitory factor [130], Adenomatosis polyposis coli downregulated 1 [131], and Tiki1 [132]. Lastly, miRNAs have been a recent area of research that have been implicated for negatively modulating Wnt signaling. Some examples include miR-200a, which decreases Wnt target gene transcription [133], miR-21, which directly represses Wnt1 protein production [134], and miR-184, which targets Wnt coreceptor FZD-7 to prevent Wnt signaling in retinal neovascularization [135].

In addition to the Notum feedback loop, there are several other negative feedback loops that regulate canonical Wnt signaling. An important one of these was mentioned in our discussion of positive Wnt regulation, with regard to the E3 ligases RNF43 and ZNRF3. These two ligases are known to control FZD membrane expression by mediating the ubiquitination of the FZD cytoplasmic loops, which leads to FZD lysosomal degradation [136, 137]. A third canonical Wnt signaling negative feedback loop is the conductin/Axin2 loop. Axin2 is a Wnt target gene, and its expression mimics Axin in the destruction complex to increase *β*-catenin degradation [138, 139], which subsequently downregulates Wnt target gene expression.

### 2.5. Iron-Mediated Wnt/*β*-Catenin Signaling during Pathological Conditions

**Figure 2:**
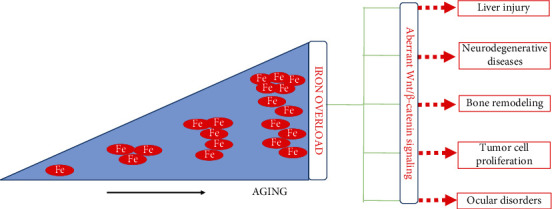
Iron accumulation associated with aging modulates canonical Wnt/*β*-catenin signaling leading to the progression of liver injury, neurodegenerative diseases, bone remodeling, cancer, and ocular disorders.

In the remainder of this review, we will discuss the recent findings on how iron modulates Wnt/*β*-catenin signaling leading to multiple disorders as outlined in Figure 2.

#### 2.5.1. Cancer

The role of Wnt signaling in cancerous pathologies is historically validated, with the discovery of Wnt signaling based on experimentation involving mouse models of breast cancer and cancer-causing retrovirus mouse mammary tumor Virus (MMTV) [140]. In experiments that lasted until the 1990s, MMTV was found to insert proviral DNA into specific regions of the mouse genome, inducing oncogene formation and mammary hyperplasia, and these genes were all later connected to Wnt signaling [141–143]. In addition, the growing clinical significance of Wnt signaling is also closely related to cancer, as the most well-known pathology that involves Wnt is familial adenomatous polyposis (FAP) [144]. In FAP, mutation in APC and stabilization of *β*-catenin results in increased colonic cell proliferation, yielding a presentation of colonic polyps associated with increased colorectal cancer risk [145–147]. In addition to MMTV studies and FAP being related to cancer, recent research has demonstrated that Wnt contributes to gastrointestinal, hematopoietic, breast, skin, brain, and colonic cancers [144].

With researchers investigating more on the connection between cancer and Wnt, iron has emerged as a prominent Wnt regulator in the context of proliferative pathologies. In studies using APC knockout cell lines Caco-2 and SW480, it was highlighted that growth on FeSO4-loaded media and hemin-loaded media, both rich in iron, increased Wnt signaling [27]. Thus, iron-mediated Wnt signaling upregulation was demonstrated in cell lines that resembled FAP-associated cancer cells with the presence of APC knockout [27]. Another recent study that investigated Nrf2 mutations and their association with hepatocellular carcinoma reported that alterations in iron homeostasis and subsequent Wnt signaling activation play a role in the occurrence and proliferation of hepatocellular carcinoma [148]. These studies show a potential contribution of iron in Wnt upregulation during cancerous pathologies.

Two recent reports revealed that iron chelators can reverse the Wnt activation during cancer. In the first study, a specific iron chelator HQBA inhibited Wnt signaling in a variety of cancer cell lines and inhibited growth of mammary tumors in MMTV-Wnt1 mouse models of Wnt-dependent breast cancer and in MMTV-PyMT mouse models of Wnt-independent breast cancer [29] indicating the plausible proliferative effects of iron both dependent and independent of Wnt signaling. The significance of this study with relation to iron is garnered from the fact that HQBA premixed with iron prior to cancer cell line exposure prevented its ability to reverse Wnt/*β*-catenin activation in several tissue-specific cancer cell lines confirming that the antitumor effect of HQBA is through iron chelation [29]. A second study identified acyl hydrazones as inhibitors of Wnt/*β*-catenin signaling by chelating iron. Upon treatment with acyl hydrazones, intracellular iron was chelated, Wnt/*β*-catenin activation was reversed, and cell proliferation significantly decreased in human CRC cell lines SW480 and DID-1 [149]. Taken together, these studies strongly suggest that excess iron upregulates Wnt/*β*-catenin signaling in certain cancerous pathologies, and that iron chelation may be a potential therapeutic strategy to prevent cancer progression.

#### 2.5.2. Neurodegenerative Disorders

The relationship between iron overload and aberrant Wnt signaling has been described in two studies that suggest excess iron in neuronal cells pathologically increases Wnt/*β*-catenin signaling. The first study discusses posthemorrhagic chronic hydrocephalus (PHCH), a potentially fatal medical condition often arising after an intraventricular hemorrhage (IVH) [150]. PHCH is known to cause an increase in both cerebral spinal fluid (CSF) iron concentration [151] and ferritin content within the brain [152]. Additionally, PHCH is characterized by fibrotic changes, which, in other tissues, are known to be linked to dysregulated Wnt/*β*-catenin signaling [153, 154]. As iron accumulation and fibrotic changes are both major players in the progression of PHCH, authors investigated the therapeutic ability of the iron chelator deferoxamine (DFX) in the treatment of abnormal Wnt signaling in a PHCH model. The study revealed that by chelating excess iron from CSF and brain ferritin, DFX normalized the upregulated Wnt/*β*-catenin signaling seen in PHCH after an IVH. This served to broadly improve PHCH occurrence and severity [155]. Similarly, another recent study reported that upregulation of Wnt/*β*-catenin signaling in neural progenitor cells (NPCs) could be normalized by DFX, leading to an increase in NPC differentiation and outgrowth [156]. These studies indicate that iron overload is detrimental to the cells in neural tissues due to Wnt/*β*-catenin activation, which can be therapeutically resolved with DFX treatment.

#### 2.5.3. Bone Remodeling

Iron [157, 158] and canonical Wnt signaling [159] are both important players in the maintenance of bone. While dietary iron is critical for healthy bone density in populations such as postmenopausal women [157], iron overload inducing diseases like *β*-thalassemia and hemochromatosis are often associated with decreased bone density and integrity [160]. Indeed, chelation of iron with deferasirox has been shown to improve bone density in patients with *β*-thalassemia [161], suggesting that excess iron is detrimental to bone health. On the other hand, normal Wnt signaling is crucial for appropriate bone remodeling because Wnt signaling is involved in both osteoblast differentiation and osteoclastogenesis [159, 162, 163]. In addition, osteoporosis, a disease characterized by reduced bone density due to ineffective bone remodeling [164], is associated with both decreased Wnt signaling and oxidative stress [165]. For these reasons, a recent article investigated iron-dependent Wnt signaling in bone marrow stromal cells differentiated towards osteoblasts [166]. The study found that excess iron is detrimental to osteoblast differentiation, and that iron chelation using DFX can reverse the negative effects of iron overload in the same cells. Moreover, the results conclude that induction of Wnt5a expression by DFX is the mediator of this recovery, which occurs through the PI3K and NFAT pathways [166]. These results indirectly indicate that excess iron reduces Wnt expression in bone; iron chelation by DFX treatment induces Wnt5a expression to recover the Wnt signaling. However, the study neither demonstrated directly that iron overload decreases Wnt signaling, nor showed that chelation of iron brings back Wnt signaling in the osteoblasts. A final consideration is that Wnt5a operates through a noncanonical Wnt pathway [167]. Despite these limitations, we can still conclude that the induction of Wnt through DFX treatment is therapeutic for iron-overloaded osteoblasts. Moreover, recent research on an *in vivo* iron-induced osteoporotic rat model demonstrated similar reduction of Wnt signaling that was recovered with DFX in bone tissues [168]. Another *in vivo* mouse study suggests that hepcidin-induced osteoporosis, mediated through iron overload, may also be targeted via inhibition of Forkhead box O3a to recover canonical Wnt signaling [169].

Interestingly, while iron overload leads to decreased activity of the Wnt pathway in osteoblasts, another study reported that iron overload induces ROS-mediated apoptosis and upregulation of the Wnt pathway in bone marrow mesenchymal cells of patients with myelodysplastic syndromes [170]. This highlights the variation in Wnt signaling in response to iron overload between different cell types even within the context of a single tissue like bone.

#### 2.5.4. Liver Injury

Chronic iron overload in hepatocytes, such as in cases of hemochromatosis, is associated with severe hepatic injury and cancer largely because of iron-induced steatosis, fibrosis, inflammation, and oxidative stress [171, 172]. Interestingly, liver-specific *β*-catenin knockout (KO) mice are known to have similar hepatic pathology resulting from factors such as fibrosis and oxidative stress [173, 174]. For these reasons, a recent study investigated the role of iron overload in hepatic pathology of liver-specific *β*-catenin KO mice [175]. This study concluded that following iron overload, liver-specific *β*-catenin KO mice have increased steatohepatitis and fibrosis that is preceded by both inflammation and oxidative stress. Moreover, they demonstrate that treatment with an antioxidant can help prevent this disease progression. In effect, the absence of *β*-catenin exacerbates the detrimental effects of hepatic iron overload. The study also showed that iron overload led to a decrease in *β*-catenin in mice where it was not knocked out. This is postulated to be an initial protective mechanism, because these mice had less expression of Cype2e1, a *β*-catenin target associated with oxidative stress [175]. However, the delicate nature of Wnt/*β*-catenin expression was proven to be detrimental in cases of *β*-catenin KO which increased inflammation and eventually lead to Cype2e1 reappearance, and, thus, further decrease in *β*-catenin due to prolonged iron overload could prove to be similarly detrimental in liver tissue. We recently showed that iron overload in normal mouse liver decreases Wnt pathway by suppressing Sirtuin 3 signaling [176]. Thus, studies so far indicate that iron overload downregulates Wnt signaling in liver contributing towards the progression of liver fibrosis.

#### 2.5.5. Ocular Diseases

Visual impairment is a national and a global health concern that impairs physical and mental health in affected individuals. The retina is one of the highest energy consuming organs in the body. Impaired metabolic changes in eye diseases often drive neuronal and vascular pathologies [177, 178]. The most prevalent retinal degenerative diseases are age-related macular degeneration and diabetic retinopathy. Identification of risk factors and the molecular mechanisms that govern neuronal cell loss and vascular changes is of interest for translational researchers and clinicians to discover preventive and interventional therapeutics for retinal disorders of the eye.

Retina expresses many iron containing proteins like RPE65, an isomerohydrolase that converts all-trans-retinyl ester to 11-cis-retinol in the visual cycle [179], fatty acid desaturase, an enzyme involved in synthesis of membrane lipids 9 [180], and guanylate cyclase, involved in the synthesis of cGMP, a second messenger in the phototransduction pathway. So iron is critical for the retinal health. However, excess iron due to its prooxidant property induces oxidative stress [181] resulting in impaired retinal function. We have previously shown that excess iron alters retinal barrier integrity and accelerates the retinal cell loss by augmenting oxidative stress and inflammasome activation [4]. Retinal iron accumulation has been reported in the human patients and mouse models of AMD and DR [4, 6]. Similarly, multiple studies have confirmed the pathogenic role of canonical Wnt signaling in the etiology of AMD [7, 8, 182] and DR [5, 183]. Recently, we reported a critical role for iron-induced oxidative stress in the activation of canonical Wnt pathway mediated by peroxisome proliferator-activated receptor- (PPAR-) alpha signaling in retina [184]. A comprehensive understanding of what drives iron overload and the downstream Wnt signaling in an increasingly wide range of diseases would help in preventing the progression of diseases at an earlier stage in the future.

## 3. Conclusion

A multivariate genomic scan has revealed high levels of iron in the blood to be intimately associated with reduced healthspan [185]. Also, intake of iron-rich diet and excess iron supplements are implicated in many age-related disorders. Iron-mediated oxidative stress induces inflammation and mitochondrial dysfunction thereby playing a critical role in the progression of cancer, neurodegenerative disorders, osteoporosis, liver fibrosis and steatosis, and ocular diseases. Similarly, aberrant changes in the canonical Wnt/*β*-catenin signaling pathway are a hallmark of cancer, diabetes mellitus, and other degenerative disorders. Elucidating the complex interplay between iron and Wnt pathway could lead to new insights into the mechanisms of disease progression and enrich our understanding of the aging biology. Control of body iron stores and Wnt inhibitors can thus serve as promising clinical targets to overcome the myriad ramifications of aging.

## Data Availability

Data supporting this Review are from previously reported studies, which have been cited.
